# Efficacy and safety analyses of epidermal growth factor receptor tyrosine kinase inhibitors combined with chemotherapy in the treatment of advanced non–small-cell lung cancer with an *EGFR/TP53* co-mutation

**DOI:** 10.1186/s12885-022-10391-z

**Published:** 2022-12-12

**Authors:** Kai Shang, Hongxiang Huang, Yongkang Xu, Yangyang Liu, Zhihui Lu, Li Chen

**Affiliations:** 1 Department of Oncology, The First Affiliated Hospital of Nanchang University, Yong-Wai Road 17, Dong-Hu District, Nanchang, 330006 China; 2 Department of Oncology, Second Affiliated Hospital of Nanchang University, Ming-De Road 1, Dong-Hu District, Nanchang, 330006 China

**Keywords:** *EGFR* mutation, *TP53* mutation, Non-small-cell lung cancer, EGFR-TKIs, Chemotherapy, Efficacy and safety analyses

## Abstract

**Purpose:**

Epidermal growth factor receptor tyrosine kinase inhibitors (EGFR-TKIs) combined with cytotoxic chemotherapy are highly effective in the treatment of advanced non–small-cell lung cancer (NSCLC) with EGFR mutations. The purpose of this study is to evaluate the efficacy and safety of this combination in advanced NSCLC patients with an *EGFR/TP53* co-mutation.

**Methods:**

Ninety-five advanced NSCLC patients with an *EGFR/TP53* co-mutation were enrolled in this study. Treatments with either EGFR-TKI monotherapy (T group, *n* = 61) or EGFR-TKI combined with chemotherapy (TC group, *n* = 34) were evaluated in relation to objective response rate (ORR), disease control rate (DCR), median time to progression (TTP), and median overall survival (OS).

**Results:**

There were no statistically significant differences in DCR between the treatment groups. The ORR was significantly improved in the TC group versus the T group (55.9% vs. 34.4%, *P* = 0.042). A higher median TTP was noted in TC group compared with T group (16.1 vs. 11.1 months, *P* = 0.002). Patients without brain metastases in TC group had a longer median OS than in T group (48.4 vs. 28.8 months, *P* = 0.003). However, there was a non-significant trend towards longer OS in TC group in the entire cohort (36.9 vs. 28.2 months, *P* = 0.078). Cox multivariate regression analysis showed that clinical stage, brain metastases, *EGFR21 L858R* mutation, and *T790M* status at first progression were independent risk factors for OS. However, the incidence of grade 3 or higher adverse events were higher in the TC group than in the T group (32.4% vs. 13.1%, *P* = 0.025).

**Conclusion:**

Our study indicates that EGFR-TKIs combined with chemotherapy could significantly improve the ORR and TTP of advanced NSCLC patients with an *EGFR/TP53* co-mutation. Combination therapy may be a promising treatment for advanced NSCLC patients with an *EGFR/TP53* co-mutation without brain metastases.

**Supplementary Information:**

The online version contains supplementary material available at 10.1186/s12885-022-10391-z.

## Introduction

Lung cancer is the one of the leading causes of cancer-related mortality and morbidity in the world, with more than 2.2 million new cases and nearly 1.8 million deaths per year [1]. Non-small cell lung cancer (NSCLC) comprises approximately 80–85% of all lung cancer types [2]. Despite major advances in the diagnosis and treatment of NSCLC, the prognosis of these patients remains poor. Indeed, more than 50% NSCLC in China presented with an advanced stage of disease at initial diagnosis, with an associated 5-year survival rate of less than 16% [3]. The discovery of EGFR-TKIs has dramatically improve the survival outcomes of advanced NSCLC patients with EGFR-TKIs sensitive mutations, and EGFR-TKIs consequently has been recommended as first-line therapy for advanced NSCLC with sensitizing EGFR mutations. The median PFS of the third-generation EGFR-TKI Osimertinib can reach 18.9 months [4]. However, the anti-tumor efficacy of TKIs varies greatly across individual patients, and correspondingly the PFS of patients treated with EGFR-TKIs ranges from several months to several years [5]. This suggests that some other factors may influence this difference of efficacy in addition to EGFR mutations. With the development of comprehensive genomic profiling, prior studies found that concomitant genetic alterations often indicate a poor prognosis compared with single-gene mutations [6].

The *TP53* gene, located in the short arm of chromosome 17 (17p13), is a tumor suppressor composed of 11 Exons [7]. *TP53* mutations are widely present in malignant tumors and are the most frequently concomitant genetic alterations in all types of lung cancer [8, 9]. The incidence of *TP53* mutations in NSCLC ranges from 30 to 60% [10]. *TP53* mutation have been shown to be negatively correlated with the prognosis of advanced NSCLC patients in numerous previous studies [11–13], and this was also verified in EGFR -mutated NSCLC patients [14, 15]. A series of recent studies also found that the concurrent mutation of *TP53* negatively affected the response to EGFR-TKIs of EGFR-mutated NSCLC [6, 16–19]. However, agents specifically target *TP53* have not been approved for NSCLC currently on the market. Improving the efficacy and survival of advanced NSCLC patients with an *EGFR/TP53* co-mutation is therefore critically important to the survival of patients with these dual mutations.

To improve the efficacy and survival of EGFR-TKIs, several combination treatments with TKIs have been evaluated in multiple retrospective studies and clinical trials [20, 21]. Some studies have shown that the combination of EGFR-TKIs and cytotoxic chemotherapy was superior to EGFR-TKIs monotherapy [22, 23]. As previous studies have analyzed the efficacy and safety of EGFR-TKIs combined with chemotherapy in EGFR mutated patients regardless of concomitant genetic alterations, the possible heterogeneity of outcomes of patients with an *EGFR/TP53* co-mutation merits further exploration.

This retrospective study intended to analyze the efficacy and safety of EGFR TKIs combined with chemotherapy in the treatment of advanced NSCLC with an *EGFR/TP53* co-mutation. We also attempted to explore the efficacy of combination therapy across different *TP53* mutation sites in order to provide reference for the clinical treatment of advanced NSCLC patients with an *EGFR/TP53* co-mutation.

## Patients and methods

### Patients

Ninety-five advanced NSCLC patients treated at The First Affiliated Hospital of Nanchang University from January 2016 to October 2020 were included. Confirmation of diagnosis was defined as on the pathologic analysis of a resected/biopsy specimen by at least two experienced pathologists at our hospital. The presence of *EGFR* and *TP53* mutations, along with their mutational status, was identified using ﻿next-generation sequencing (NGS) using tumor tissue samples or peripheral blood cell-free tumor DNA (ctDNA) at our hospital or the referring institution [24]. Other inclusion criteria containing an Eastern Cooperative Oncology Group performance status (ECOG PS) of 0–2 and first-line therapy with either EGFR-TKI monotherapy (T) or EGFR-TKI combined with pemetrexed based chemotherapy (TC). The exclusion criteria were as follows: older than 75 years old or less than 18 years old; primary organs failure; accompanied with other malignancies; unable to follow-up; and less than two cycles of chemotherapy. A review of the mutational features of each gene was performed. The clinical data and medical course of each patient were collected via retrospective analysis of inpatient medical records. Pathological classification of tumor was based on The World Health Organization (2015 edition) pulmonary tumor tissue type [25]. Clinical stage at the time of EGFR-TKI treatment was classified according to the American Joint Commission on Cancer (AJCC), 8th Edition tumor-node-metastases staging system [26].

### Treatment methods

Patients in the TC group were given EGFR-TKIs (gefitinib 250 mg once daily (qd), or erlotinib 150 mg qd, or icotinib 125 mg three times a day, or osimertinib 80 mg qd) combined with pemetrexed (500 mg/m2) based chemotherapy (mainly pemetrexed plus platinum). Treatment continued until disease progression or unacceptable toxicity. Given the intensity of the treatment-associated side effects, short-term dose adjustments or delays were allowed according to the individual response to chemotherapy, and the specific number of chemotherapy cycles could be adjusted according to the efficacy of the drug and the tolerance of the patient. Some patients also received concurrent EGFR-TKIs and pemetrexed maintenance after 6 cycles of chemotherapy. Patients in the T group took EGFR-TKIs monotherapy.

### Response evaluation

Treatment evaluation were conducted in all patients receiving either T or TC. Therapeutic efficacy was measured every 6–8 weeks from the beginning of EGFR-TKIs treatment in accordance with the Response Evaluation Criteria in Solid Tumors (RECIST, version 1.1) using computed tomography (CT) scans [27]. Tumor response including complete response (CR), partial response (PR), stable disease (SD), or progressive disease (PD). Tumor response rate was expressed with objective response rate (ORR) and disease control rate (DCR). ORR was defined as the percentage of patients who achieved CR or PR, while DCR was defined as the percentage of patients who achieved CR, PR or SD. Time to progression (TPP) was defined as the interval from the initiation of EGFR-TKIs treatment to disease progression. Overall survival (OS) was calculated from the date of receiving EGFR-TKIs treatment to the date of cancer-related death, or the last day of follow-up. Adverse events (AEs) were evaluated according to the Common Terminology Criteria for Adverse Events 5.0 (CTCAE 5.0). The follow-up time of each patient was calculated from the beginning of treatment to the time of the relevant end point or the date of the most recent patient follow-up evaluation. The data deadline was October 31, 2021, with a median follow-up time of 25.2 months (8.4 months to 67.0 months).

### Statistics

Statistical analyses of baseline characteristics were performed using Pearson’s χ2 test or Fisher’s exact test. TTP and OS were calculated using the Kaplan–Meier method, and log-rank test was used for comparison between groups. Independent factors associated with TTP and OS were calculated using univariate and multivariate cox regression models. All statistical analyses tests were performed using SPSS 26.0 software and R software version 4.0.3, *P* < 0.05 was considered statistically significant.

## Results

### Patient characteristics

**Fig. 1:**
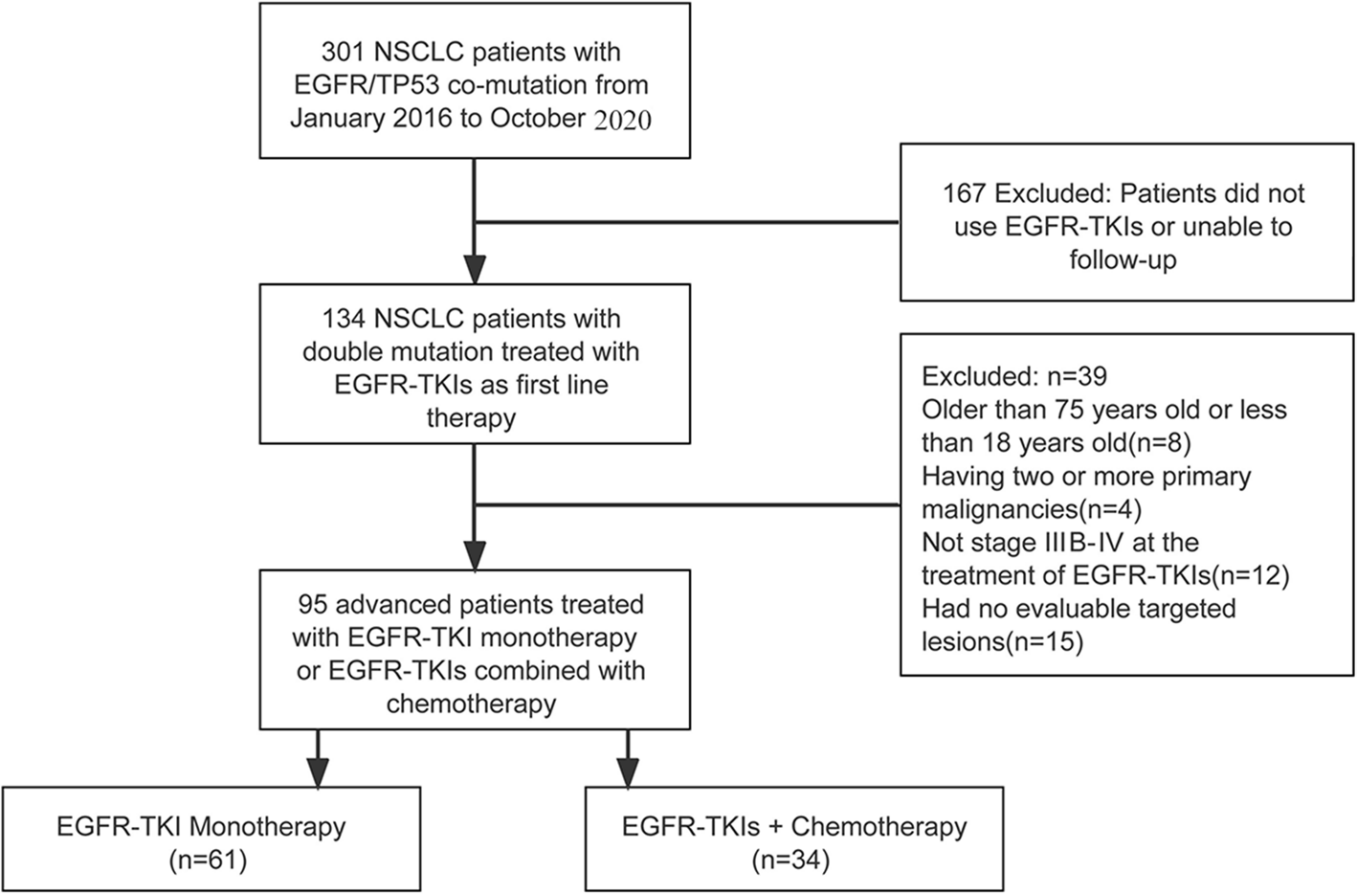
Flowchart of the patients included in the present study

A total of 301 patients were diagnosed with advanced NSCLC with *EGFR/TP53* dual mutations from January 2016 to October 2020 at the First Affiliated Hospital of Nanchang University. Of these, 134 patients met our inclusion criteria, and 95 NSCLC patients treated with T or TC were ultimately enrolled (Fig. 1).

The baseline characteristics of the included patients are shown in Table 1. Sixty-one NSCLC patients received EGFR-TKI monotherapy, and the other 34 received EGFR-TKIs combined with pemetrexed based chemotherapy. Most patients (28/34) in the TC group received 4–6 cycles of chemotherapy. The average number of chemotherapy cycles was 5.6. It is worth noting that a 58-year-old patient received 17 cycles of pemetrexed maintenance after 6 cycles of chemotherapy. *T790M* mutation status at first progression was evaluated in 68 patients treated with first generation EGFR-TKIs in both groups using tumor tissue samples or peripheral blood cell-free tumor DNA, forty-four (64.7%) patients were finally confirmed to have a *T790M* mutation at their first progression. The *T790M* mutation status of 27 patients was unknown or untested. Post-treatments after progression were showed in Supplementary Table S1. 59% (36/61) of the patients in the T group and 47% (16/34) patients in the TC group received third-generation EGFR-TKI (mainly osimertinib) treatment after the first TKI progression, which was not statistically significant (*P* = 0.262).

**Table 1:** Patient baseline characteristics (*n* = 95)

Characteristic	T group (*n* = 61)	TC group (*n* = 34)	*P*
Gender			0.110
Male	22(36.07)	18(52.94)	
Female	39(63.93)	16(47.06)	
Age			0.440
< 60	39 (63.93)	19 (55.88)	
≥ 60	22 (36.07)	15 (44.12)	
Smoking history			0.949
No	47 (77.05)	26 (76.47)	
Yes	14 (22.95)	8 (23.53)	
ECOG PS			0.646
0–1	58 (95.08)	33 (97.06)	
2	3 (4.92)	1 (2.94)	
Histology			0.257
Adenocarcinoma	60 (98.36)	32 (94.12)	
Non-adenocarcinoma	1 (1.64)	2 (5.88)	
Clinical Stage			0.719
IIIB/C	9 (14.75)	6 (17.65)	
IV	34 (55.74)	16 (47.06)	
IV with BM	18 (29.51)	12 (35.29)	
Types of EGFR-TKIs			0.557
Gefitinib	41 (67.20)	26 (76.47)	
Icotinib	13 (21.31)	5 (14.71)	
Erlotinib	2 (3.29)	2 (5.88)	
Osimertinib	5 (8.20)	1 (2.94)	
T790M status			0.686
Negative	17 (27.87)	7 (20.59)	
Positive	28 (45.90)	16 (47.06)	
Unknow	16 (26.23)	11 (32.35)	
EGFR mutation			0.471
EGFR19 Del	32(52.46)	16(47.06)	
EGFR21 L858R	22(36.07)	16(47.06)	
Other mutations	7(11.47)	2(5.88)	
TP53 mutation			0.810
Exon4	6(9.83)	1(2.94)	
Exon5	17(27.87)	11(32.35)	
Exon6	4(6.57)	2(5.88)	
Exon7	14(22.95)	10(29.41)	
Exon8	12(19.67)	5(14.71)	
Other mutations	8(13.11)	5(14.71)	

*BM* brain metastases, *T790M status* T790M mutation status at first progression

**Fig. 2:**
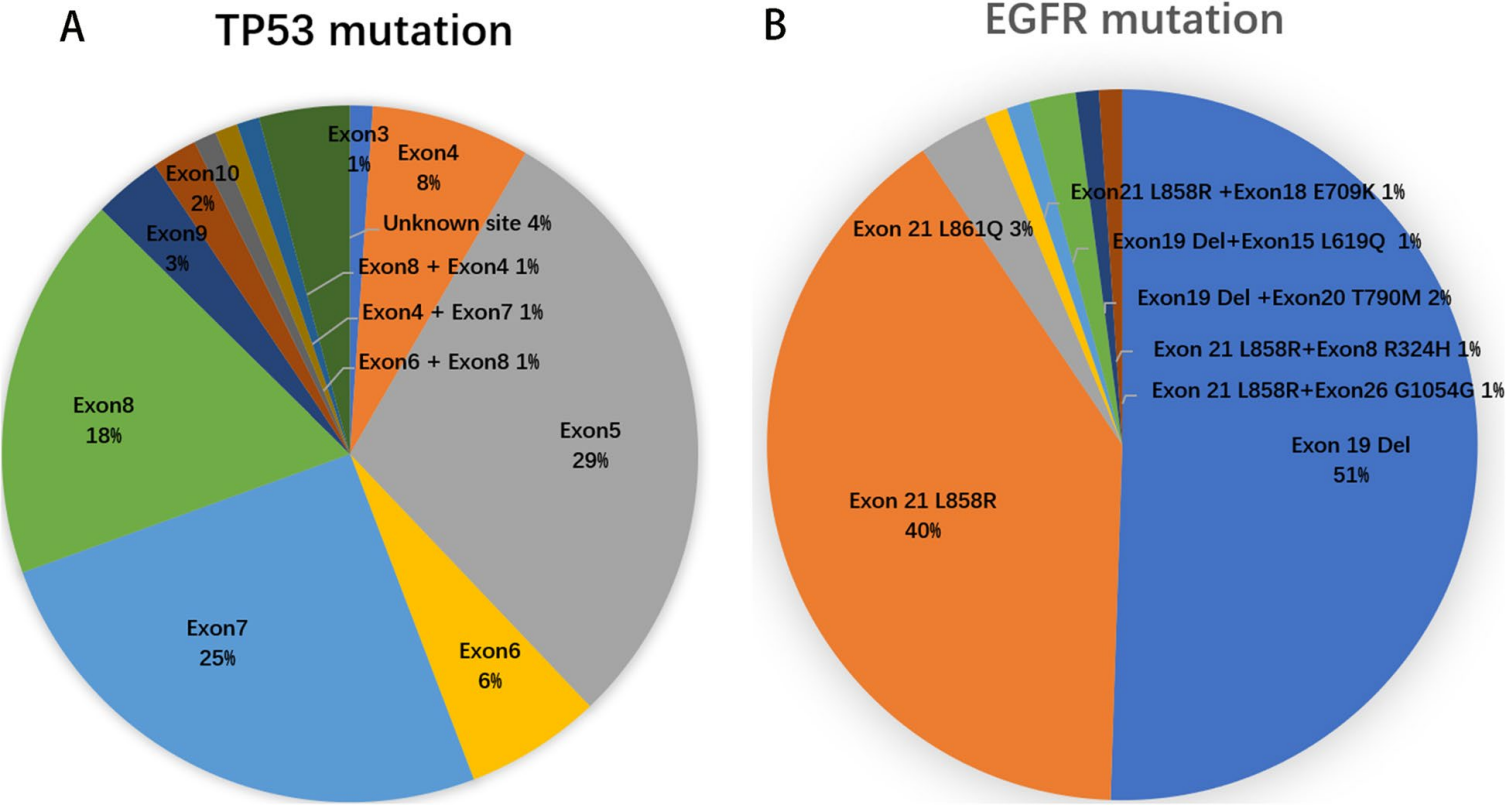
Distribution of *TP53* mutation (**A**) and *EGFR* mutation (**B**) sites in patients with a co-mutation

All 95 patients carried *EGFR/TP53* double mutations. *TP53* mutations mainly occurred at Exon5-Exon8 (78.95%), 7 mutations were in exon 4 (7.37%), 28 in exon 5 (29.47%), 6 in exon 6 (6.32%), 24 in exon 7 (25.26%), 17 in exon 8 (17.89%), 3 in exon 9 (3.16%), 2 in exon 10 (2.11%) and 4 (4.22%) at an unknown site. A single rare case of an exon 3 R77L mutation was recorded. Three cases with multiple mutations were also included in our study (Exon6 H193Y + Exon8 G279E, Exon4 D49Vfs*4 + Exon7 S241F and Exon8 E294X + Exon4 W53X) (Fig. 2A). EGFR mutations mainly occurred as exon 19 deletions (50.53%) and exon 21 mutations (40%, 41/95; 38 L858R, 3 L861Q). There were 6 cases of double *EGFR* mutations (1 Exon21 L858R + Exon18 E709K, 1 Exon19 Del + Exon15 L619Q, 1 Exon 21 L858R + Exon8 R324H, 1 Exon 21 L858R + Exon26 G1054G, and 2 Exon19 Del + Exon20 T790M) (Fig. 2B).

### Clinical efficacy

**Fig. 3:**
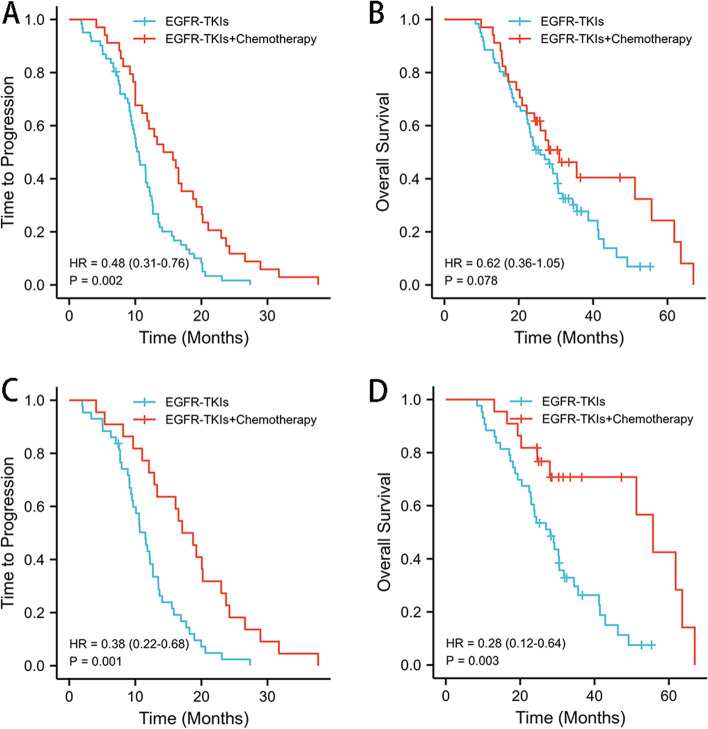
TTP (**A**) and OS (**B**) curves of *EGFR/TP53* co-mutation patients receiving EGFR-TKI monotherapy (T) or EGFR-TKIs combined with chemotherapy (TC). TTP (**C**) and OS (**D**) curves of *EGFR/TP53* co-mutation patients without brain metastases receiving T or TC

Therapeutic efficacy is summarized in Table 2. No patients achieved CR in both arms, there are more patients achieve PR in TC group than T group (55.9% vs. 34.4%, *P* = 0.042).SD was observed in 35 patients in T group and 14 patients in TC group. The ORR of the TC group (55.9%) was higher than that of the T group (34.4%). The DCR of the T group was 91.8% (56/61), and the DCR of the TC group was 97.1% (33/34). While the 1-year OS (94.1%) and 2-year OS (64.7%) of the TC group was higher, differences were not statistically significant. The median TTP of the T group was 11.1 months (95% CI: 9.719–12.400), and that of the TC group was 16.1 months (95% CI:13.392–18.722, *P* = 0.002, Fig. 3A). While the median OS of the T group was 28.2 months (95% CI: 24.716–31.734), and that of the TC group was 36.9 months (95% CI:29.323–44.510), this difference was not statistically significant (*P* = 0.078, Fig. 3B). When patients with brain metastases were excluded, the median TTP of the TC group (18.2 months, 95% CI:14.628–21.772) was longer than the T group (11.6 months, 95% CI:10.002–13.262) (*P* = 0.001, Fig. 3C). OS differences between the TC group (48.4 months, 95% CI:39.492–57.405) and the T group (28.8 months, 95% CI:24.685–32.952) were also statistically significant (*P* = 0.003, Fig. 3D).

**Table 2:** *EGFR/TP53* co-mutation patients treatment outcomes

	T group n (%)	TC group n (%)	*P*
Response			
CR	0	0	
PR	21(34.4)	19(55.9)	0.042
SD	35(57.4)	14(41.2)	0.130
PD	5(8.2)	1(2.9)	0.313
ORR	21(34.4)	19(55.9)	0.042
DCR	56(91.8)	33(97.1)	0.313
TTP			
6 months progression free	52(85.2)	31(91.2)	0.404
12 months progression free	22(36.1)	23(67.6)	0.003
18 months progression free	8(13.1)	12(35.3)	0.011
OS			
1-year OS	54(88.5)	32(94.1)	0.372
2-year OS	32(52.5)	22(64.7)	0.248

**Fig. 4:**
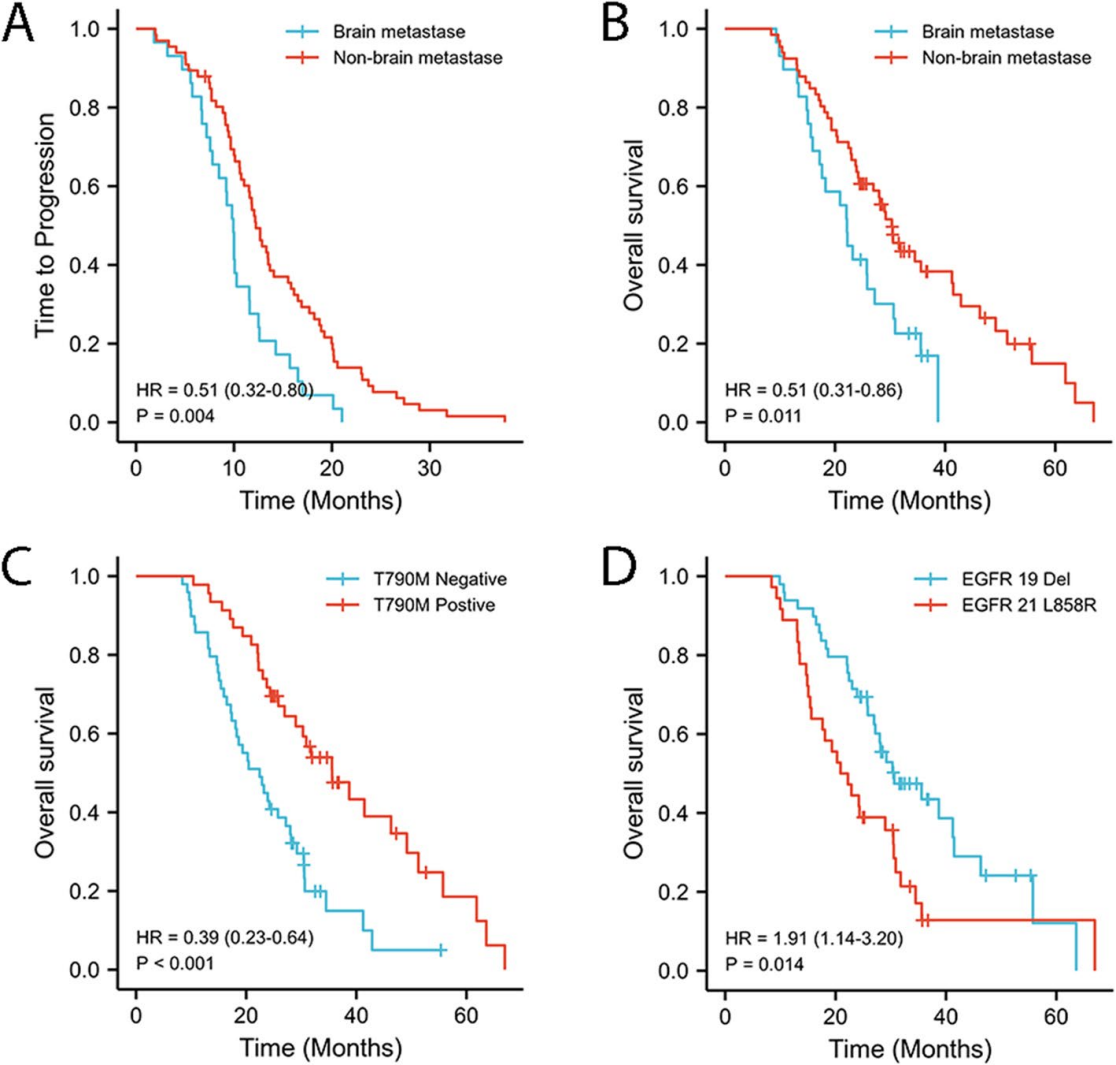
TTP (**A**) curves of *EGFR/TP53* co-mutation patients divided by the presence of brain metastases; **B**, **C**, **D** OS curves of *EGFR/TP53* co-mutation patients stratified by the presence of brain metastases (**B**), *T790M* status at progression (**C**) and *EGFR* mutation subtypes (**D**)

The two subgroups of *EGFR/TP53* co-mutation patients stratified according to the presence of brain metastases showed obvious differences in TTP (*P* = 0.004, Fig. 4A) and OS (*P* = 0.011, Fig. 4B). Similar subgroups stratified according to *T790M* status at progression also had significant different OS (*P* < 0.001, Fig. 4C).Otherwise, subgroups divided by *EGFR* exon 19 deletions or exon 21 L858R mutations had statistically different OS (*P* = 0.014, Fig. 4D). We also tried to clarify the relationship between *TP53* mutation site with TTP and OS. However, no significant differences were observed.

**Fig. 5:**
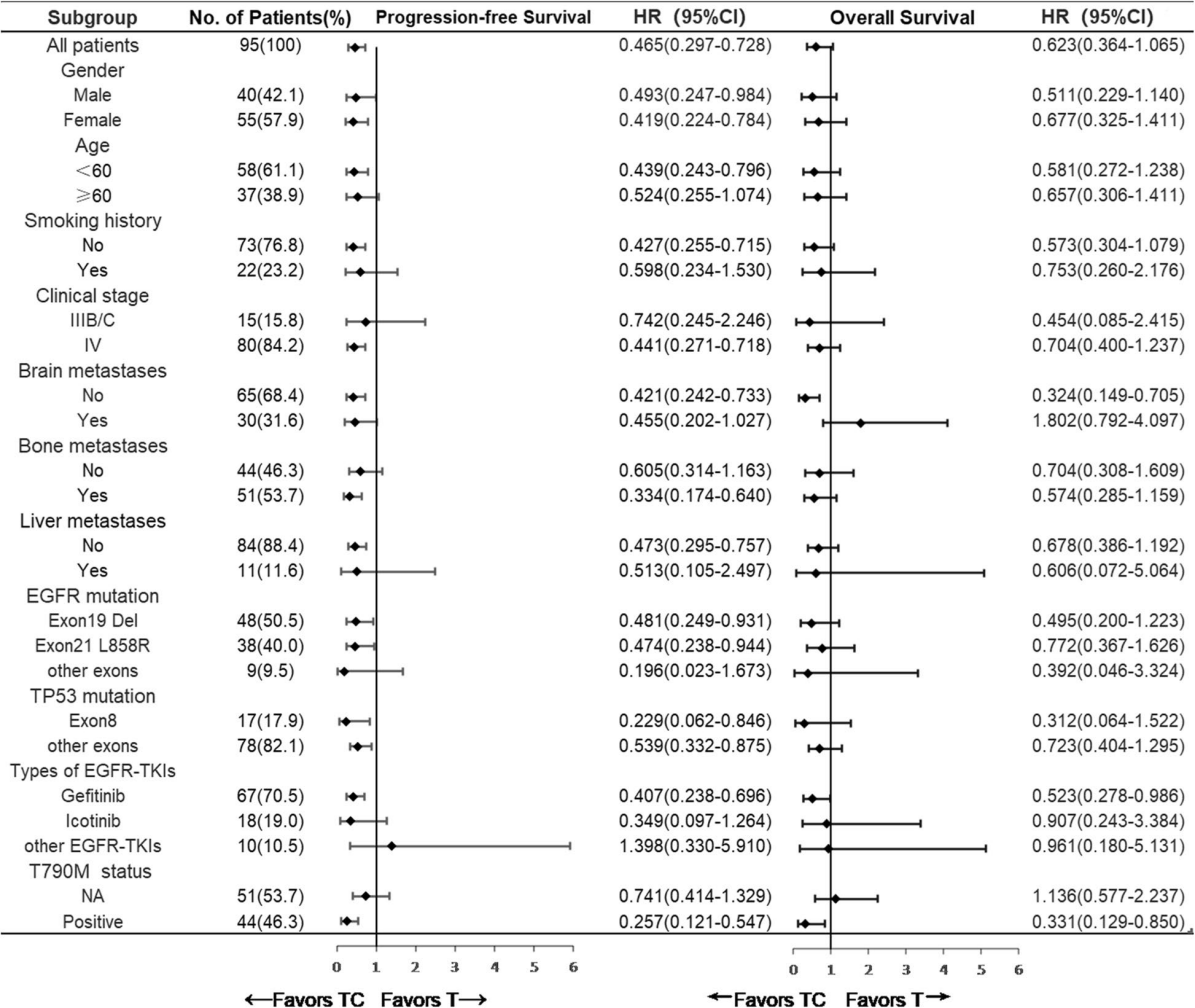
Forest plot for TTP and OS of *EGFR/TP53* co-mutation patients treated with T or TC

Forest plot analysis shows factors associated with TTP and OS (Fig. 5). Patients with T790M mutation at first progression or without brain metastases may benefit better from TC treatment. There are no treatments differences in both groups of patients with different EGFR or TP53 mutations.

### Safety

Adverse events of all grades related to treatment occurred in 70.6% (24/34) of the patients in the TC group and 59.0% (36/61) of patients in the T group, with no significant difference (*P* = 0.262). The most common AEs of all grades in the TC group including rash (55.6%), leukopenia (52.9%), thrombocytopenia (50%), neutropenia (47.1%), anemia (47.1%), and liver dysfunction (32.3%). While the most common AEs in the T group were rash (37.7%), nail changes (32.8%), diarrhea (21.3%) and anemia (19.7%). The majority of patients experienced grade 1–2 AEs during treatment period, but the rate of grade ≥ 3 treatment-related AEs in the TC group was significantly higher than that in the T group (32.4% vs. 13.1%, *P* = 0.025). Compared with the T group, the significantly increased AEs of grade ≥ 3 in the TC group were mainly related to bone marrow suppression including leukopenia (8.9% vs. 0), neutropenia (14.7% vs. 0), anemia (8.9% vs. 3.3%), and thrombocytopenia (8.9% vs. 0). Most patients had a good tolerance in both groups, one patient in the T group had a short-term suspension due to sever rash, several patients in the TC group successfully completed 4–6 cycles of chemotherapy after short-lived delays or adjusting the dose of chemotherapy drugs. No treatment-related deaths occurred during the whole treatment. (Table 3).

**Table 3:** Common adverse events

Adverse Events	All Grades			Grade ≥ 3		
	TC group (*n* = 34)	T group (*n* = 61)	*P*	TC group (*n* = 34)	T group (*n* = 61)	*P*
Any TEAE (≥ 1)	24(70.6)	36(59.0)	0.262	11(32.4)	8(13.1)	0.025
Leukopenia	18(52.9)	2(3.3)	*P* < 0.001	3(8.9)	0(0.0)	0.018
Neutropenia	16(47.1)	3(4.9)	*P* < 0.001	5(14.7)	0(0.0)	0.002
Anemia	16(47.1)	12(19.7)	0.005	3(8.9)	2(3.3)	*P* < 0.001
Thrombocytopenia	17(50.0)	5(8.2)	*P* < 0.001	3(8.9)	0(0.0)	0.018
Liver dysfunction	11(32.3)	9(14.8)	0.044	2(5.8)	2(3.3)	0.545
Creatinine elevation	5(14.7)	4(6.6)	0.194	0(0.0)	0(0.0)	
Diarrhea	9(26.5)	13(21.3)	0.568	1(2.9)	1(1.6)	0.672
Vomiting	7(20.6)	2(3.3)	0.006	0(0.0)	0(0.0)	
Stomatitis	8(23.5)	11(18.0)	0.521	0(0.0)	0(0.0)	
Rash	19(55.6)	23(37.7)	0.087	4(11.7)	5(8.2)	0.569
Nail changes	7(20.6)	20(32.8)	0.268	1(2.9)	0(0.0)	0.178
Constipation	10(29.4)	6(10.0)	0.007	0(0.0)	0(0.0)	

*TEAE* treatment-emergent adverse event

### Prognostic factor analysis

Univariate and multivariate Cox regression models of TTP and OS were performed using the clinical and molecular parameters of patients with *EGFR/TP53* co-mutations. Both univariate and multivariate analysis showed brain metastases and EGFR-TKI monotherapy were statistically valuable significant predictors of a poor TTP (*P* = 0.006 and *P* = 0.001, *P* = 0.002 and *P* < 0.001, respectively). Clinical stage, brain metastases, bone metastases, liver metastases, *EGFR 21 L858R* mutation and *T790M* status at progression may affect OS time. Multivariate analysis identified brain metastases, advanced clinical stage and EGFR exon21 L858R mutation were related to a worse OS, while *T790M* positive at progression may herald a better one (Table 4).

**Table 4:** Univariate and multivariate cox regression analyses

Variables	TTP					OS			
	Univariate		Multivariate			Univariate		Multivariate	
	HR (95% Cl)	*P*	HR (95% Cl)	*P*		HR (95% Cl)	*P*	HR (95% Cl)	*P*
Gender (Male vs. Female)	0.956(0.634–1.441)	0.830			0.821(0.507–1.328)		0.421		
Age (< 60 vs. ≥ 60)	0.905(0.595–1.377)	0.642			1.583(0.979–2.559)		0.061		
Smoking status (No vs. Yes)	1.333(0.820–2.165)	0.246			1.472(0.846–2.560)		0.171		
ECOG PS (0–1 vs. 2)	2.694(0.974–7.455)	0.056			1.429(0.445–4.590)		0.549		
Clinical stage (IIIB/C vs. IV)	1.188(0.682–2.069)	0.543			3.135(1.417–6.935)		0.005	2.640(1.061–6.564)	0.037
Brain metastases (No vs. Yes)	1.892(1.200–2.984)	0.006	2.065(1.305–3.269)	0.002	1.950(1.162–3.270)		0.011	2.136(1.207–3.781)	0.009
Bone metastases (No vs. Yes)	1.100(0.731–1.654)	0.648			1.796(1.106–2.918)		0.018	1.517(0.892–2.580)	0.124
Liver metastases (No vs. Yes)	1.256(0.665–2.372)	0.482			2.171(1.096–4.301)		0.026	1.365(0.666–2.798)	0.396
EGFR 21 L858R mutation (No vs. Yes)	0.903(0.734–1.111)	0.333			0.774 (0.610–0.982)		0.035	2.221(1.314–3.752)	0.003
TP53 mutation (Exon8 vs. Other Exons)	0.835(0.490–1.424)	0.509			0.795 (0.431–1.465)		0.462		
T790M status at progression (NA vs. Positive)	0.673(0.445–1.018)	0.061			0.378(0.227–0.631)		*P* < 0.001	0.294(0.169–0.510)	P < 0.001
Treatment (T vs. TC)	0.465(0.297–0.728)	0.001	0.438(0.279–0.687)	*P* < 0.001	0.623 (0.364–1.065)		0.084		

*HR* hazard ratio, *CI* confidence interval, *NA* unknown or unable to perform check calculation

## Discussion

*EGFR/TP53* co-mutation makes NSCLC patients resistant to EGFR-TKIs and chemotherapy, thus shortening PFS and reducing OS [10, 28]. However, the mechanisms and the mitigation of this co-mutation on the efficacy and prognosis of NSCLC remains unclear. Here, we investigated the efficacy and safety of EGFR-TKIs combined with chemotherapy in the treatment of patients with an *EGFR/TP53* co-mutation.

The *TP53* gene consists of 11 exons, and mutation hotspots were found to be concentrated in exons 5–8. In our case series, we found that 78.95% of patients carried a *TP53* mutation in exons 5–8, and that exon 5 and exon 7 were the most frequent mutation sites (accounting for 29.47% and 25.26% of mutations respectively), which was consistent with prior work [29]. It is well acknowledged that different mutation sites may bring changes in the *p53* protein, which mainly have diverse different biological significance [30, 31]. Mutations in the DNA-binding domain (exons 5–8) may endue *p*53 protein with gain-of-function properties, resulting in the pro-oncogenic features of the *TP53* [32]. A recent study found that patients with a *TP53* exon 8 mutation had a worse disease control rate than wild-type patients (41.7% vs. 87.3%, *P* < 0.001) treated with EGFR-TKIs in the first line setting, and that the risk of disease progression in patients with a *TP53* exon 8 mutation was almost 10 times over that of wild-type patients (*P* < 0.05).The study also found *TP53* exon 8 mutation was associated with a significantly shorter progression-free survival (PFS) [16]. Few studies have reported the relationship between *TP53* mutation and the efficacy of EGFR-TKIs plus chemotherapy. Yang found that concurrent *TP53* mutations, especially exon 4 and 6, were associated with a shorter TTP on EGFR-TKI monotherapy (11.4 months vs. 16.6 months, *P* = 0.003), while EGFR–TKIs plus chemotherapy would benefit those patients more (with *TP53*: 11.4 months vs. 19.1 months, *P* = 0.001, HR = 0.407; without *TP53*: 16.6 months vs. 18.9 months, *P* = 0.379, HR = 0.706) [33]. Unfortunately, we did not include *TP53* wild-type patients in our study, whether the improved efficacy of EGFR-TKIs plus chemotherapy was related to the presence of p53 still remains unknown. In addition, a secondary analysis of phase III clinical study CTONG 0901 found that exon 4 or 7 mutation of *TP53* were independent prognostic factors for shortened PFS and OS in patients with EGFR-mutant advanced NSCLC. The median PFS in patients with mutations in exon 4 or 7 of *TP53*, other *TP53* mutations, and wild-type *TP53* were 9.4, 11.0, and 14.5 months, respectively (*P* = 0.009), and median OS were 15.8, 20.0, and 26.1 months, respectively (*P* = 0.004) [34]. We also sought to find differences in prognosis of EGFR mutated patients with different *TP53* mutation sites or mutation types, no differences were observed in treatment outcomes in both groups (Supplementary Table S2, Supplementary Figure S1 and Figure S2). The reasons may be multi-faceted. Firstly, most studies compare patients of different *TP53* mutation sites with *TP53* wild-type patients, while all the patients in our study are with *EGFP/TP53* co-mutation. Secondly, there were differences in patients' baseline, treatment regimens, and sample size. Therefore, the impact of *TP53* mutation sites or types on the prognosis of patients with *EGFR/TP53* co-mutation remains to be further explored.

In order to improve the efficacy and survival of *EGFR/TP53* co-mutation patients, effective treatment options should be identified. EGFR-TKIs have been regarded as the gold standard for advanced NSCLC patients with *EGFR* sensitive mutations. However, the efficacy of EGFR-TKI monotherapy is not ideal, and the majority of patients will develop drug resistance after 12 -18 months of treatment [5]. In order to overcome drug resistance to EGFR-TKIs and further improve their clinical efficacy, combining EGFR-TKIs with conventional chemotherapy has gradually attracted more attention [35–37]. Studies have shown that the combination of gefitinib or afatinib with pemetrexed can produce synergistic anti-proliferation and pro-apoptosis effects on NSCLC cell lines in vitro, which subsequently inhibits TKI resistance [38, 39]. Several clinical studies have also fully demonstrated the great advantages of combined therapy: in the phase II randomized controlled JMIT study, PFS in patients treated with gefitinib combined with pemetrexed was significantly improved than patients treated with gefitinib monotherapy (15.8 vs. 10.9 months, respectively, *P* < 0.001) [40]. Another phase-III clinical study NEJ009 found that pemetrexed + carboplatin chemotherapy combined with gefitinib could lead to improved PFS (20.9 vs. 11.9 months, *P* < 0.001) and OS (50.9 vs. 38.8 months, *P* < 0.021) compared with gefitinib alone. The PFS of the combination group even exceeded 18.9 months when using Osimertinib as first-line treatment in the FLAURA study [22]. Gefitinib or erlotinib combined with chemotherapy has been recommended as a first-line treatment for stage IV EGFR mutant NSCLC (PS = 0–1) in the NSCLC guidelines of Chinese Society of Clinical Oncology in 2017. Based on these results, we compared the efficacy and safety of EGFR-TKIs combined with chemotherapy or EGFR-TKIs in the setting of an *EGFR/TP53* co-mutation.

This study included 95 clinical cases, and compared real-world survival data of patients treated with either T or TC. Its short-term efficacy results showed that the ORR in the TC and T groups were 55.9% (19 / 34) and 34.4% (21 / 61), respectively. The addition of chemotherapy can significantly improve the efficacy of EGFR-TKIs in patients with a co-mutation (*P* = 0.042). There was no significant difference in DCR between the two groups (*P* = 0.313). Long-term efficacy results showed that compared with T, TC can prolong the median TTP to a certain extent (16.1 vs. 11.1 months, *P* = 0.002). The median TTP in the combination group was lower than the median PFS published in several similar clinical trials [22, 37], which may be related to many factors including ethnic differences, tumor load, targeted drugs, gene mutation states and patient compliance problems. The median TTP of the monotherapy group was basically the same as that of these studies. We also found that the addition of chemotherapy prolonged overall survival of *EGFR/TP53* co-mutation patients, but this was not significant (36.9 vs. 28.2 months, *P* = 0.078). There are several possible reasons why the addition of chemotherapy to EGFR-TKIs did not significantly improve the overall survival. First, treatment after disease progression may affect overall survival. Due to the increasing number of treatment options available for NSCLC, the impact of first-line treatment on overall survival may be skewed by subsequent therapies [41, 42]. We also recognized the existence of selection bias that may affect treatment outcomes. However, we did find that EGFR-TKIs plus chemotherapy could significantly improve the OS of *EGFR/TP53* co-mutation patients without brain metastases (48.4 vs. 28.8 months, *P* = 0.003). This may because most chemotherapy drugs cannot cross the blood–brain barrier, and therefore the effects of chemotherapy on brain tumors are disappointing. Our results suggest that TC may be the promising treatment for *EGFR/TP53* co-mutation advanced NSCLC patients who have no brain metastases.

Factors affecting TTP and OS were also analyzed. Combination therapy was an independent protective factor against disease progression (HR = 0.438,95% CI: 0.279–0.687, *P* < 0.001), while the presence of brain metastases was an independent risk factor for disease progression (HR = 2.065,95% CI: 1.305–3.269, *P* = 0.002). Our study shows that during the population of *EGFR/TP53* co-mutation NSCLC, patients without brain metastases may benefit better from combination therapy. However, our conclusions need to be further validated in large randomized clinical studies, it is necessary to design a prospective study to figure out the best beneficiaries from the combination of EGFR-TKIs and pemetrexed based chemotherapy. Moreover, brain metastases and *EGFR21 L858R* mutation were also independent risk factors for a shorter OS of EGFR/TP53 co-mutation patients (HR = 2.136,95% CI:1.207–3.781, *P* = 0.009; HR = 2.221,95% CI:1.314–3.752, *P* = 0.003), while T790M positive status at progression was an independent protective factor (HR = 0.294, 95% CI: 0.169–0.510, *P* < 0.001). Liver metastasis has been shown to be a poor prognostic factor in advanced NSCLC patients who received cytotoxic chemotherapy or targeted therapy in a series of previous studies. However, liver metastasis was not found to be an independent risk factor in our study, this may be highly correlated with the sample size, patient baseline level or other concerning factors. To our knowledge, whether the presence of *p53* is correlated with the prognosis of patients with liver metastases in non-small cell lung cancer still remains to be furthered explored. It is worth noting that patients with *EGFR21 L858R* mutation had worse clinical benefit in overall survival than those with an *EGFR19 Del* mutation, which is consistent with the current literature [43]. However, no effects on PFS and OS were observed in clinical trials such as IPASS [44] and NEJ002 [45]. Whether the overall survival of *EGFR/TP53* co-mutation patients is related to the *EGFR* mutation status remains to be further discussed. We also noticed that patients who were *T790M* positive at first progression obviously had an improved OS. This may mainly because those patients could be continually treated with third generation EGFR-TKIs.

In terms of drug safety, large randomized clinical studies such as NEJ005, JMIT, and NEJ009 have fully demonstrated that EGFR-TKIs combined with chemotherapy is generally safe and tolerant compared with EGFR-TKIs monotherapy, combination therapy does not significantly increase the frequency and severity of adverse reactions at all levels. The safety findings of our study showed that the incidence of grade 3 or above AEs in the TC group was higher than T group (32.4% vs.13.1%, *P* = 0.025), but there was no statistic difference in the incidence of all grade AEs between the two groups (70.6% vs. 59.0%, *P* = 0.262). Compared with the monotherapy group, the addition of chemotherapy mainly increases the risk of medulla regression and gastrointestinal reactions (*P* < 0.05). The results of this study are mainly consistent with those of prior works [46]. As expected, the increased incidence of AEs related to bone marrow suppression and the digestive tract were primarily related to the toxic reactions of pemetrexed and platinum. However, the majority of patients have a good tolerance.

In conclusion, our study shows that the combination of EGFR-TKIs and pemetrexed based chemotherapy could significantly improve the ORR and TTP of advanced NSCLC patients with an *EGFR/TP53* co-mutation compared with EGFR-TKI monotherapy. In patients without brain metastases, EGFR-TKIs combined with chemotherapy has better efficacy and controllable safety in the first line treatment. Combination therapy may therefore be an alternative treatment for *EGFR/TP53* co-mutation advanced NSCLC patients. Although the concomitant use of chemotherapy can potentially increase the risk of adverse effects, most side effects were generally manageable without an emergent safety concern. However, the present work has a limited sample size, and some patients had not reached their end point. In addition to this, the information about post-treatments after progression of some patients in our study is incomplete, patients’ OS may also be affected by the regimens of post-treatment after disease progression. These research results can therefore be improved by expanding the number of samples and prolonging the follow-up time. This study provides a certain clinical reference basis for EGFR-TKIs combined with chemotherapy in the treatment of advanced *EGFR/TP53* co-mutation advanced NSCLC.

## Supplementary Information

**Additional file 1: Supplementary Table S1.** Post-treatments after progression in T and TC group (*n*=95). **Supplementary Table S2.** TP53 mutations classified by missense or nonsense mutations; **Supplementary Figure S1.** TTP (A) and OS (B) curves of EGFR/TP53 co-mutation patients clarified by missense mutations or nonsense mutations. **Supplementary Figure S2.** TTP (A) and OS (B) curves of EGFR/TP53 co-mutation patients clarified by missense mutations or nonsense mutations in T group. TTP (C) and OS (D) curves of EGFR/TP53 co-mutation patients clarified by missense mutations or nonsense mutations in TC group.

## Data Availability

The raw data presented in this study were included in the article and further inquiries can be directed to the corresponding author.

## Abbreviations

EGFR-TKIs: Epidermal growth factor receptor tyrosine kinase inhibitors
NSCLC: Non–small-cell lung cancer
ORR: Objective response rate
DCR: Disease control rate
TTP: Median time to progression
OS: And median overall survival
NGS: Next-generation sequencing
ctDNA: Cell-free tumor DNA
ECOG PS: Eastern Cooperative Oncology Group performance status
T: EGFR-TKI monotherapy
TC: EGFR-TKI combined with pemetrexed based chemotherapy
AJCC: American Joint Commission on Cancer
RECIST: Response Evaluation Criteria in Solid Tumors
CR: Complete response
PR: Partial response: SD: stable disease
PD: Progressive disease
AEs: Adverse events
CTCAE: Common Terminology Criteria for Adverse Events

## References

[1] Sung H, Ferlay J, Siegel RL (2021). Global Cancer Statistics 2020: GLOBOCAN Estimates of Incidence and Mortality Worldwide for 36 Cancers in 185 Countries. CA Cancer J Clin.

[2] Bray F, Ferlay J, Soerjomataram I, Siegel RL, Torre LA, Jemal A (2018). Global cancer statistics 2018: GLOBOCAN estimates of incidence and mortality worldwide for 36 cancers in 185 countries. CA Cancer J Clin.

[3] Rosell R, Karachaliou N (2016). Large-scale screening for somatic mutations in lung cancer. Lancet.

[4] Soria JC, Ohe Y, Vansteenkiste J (2018). Osimertinib in Untreated EGFR-Mutated Advanced Non-Small-Cell Lung Cancer. N Engl J Med.

[5] Yu HA, Arcila ME, Rekhtman N (2013). Analysis of tumor specimens at the time of acquired resistance to EGFR-TKI therapy in 155 patients with EGFR-mutant lung cancers. Clin Cancer Res.

[6] Kim Y, Lee B, Shim JH (2019). Concurrent Genetic Alterations Predict the Progression to Target Therapy in EGFR-Mutated Advanced NSCLC. J Thorac Oncol.

[7] Hainaut P, Hollstein M (2000). p53 and human cancer: the first ten thousand mutations. Adv Cancer Res.

[8] Cancer Genome Atlas Research Network (2012). Comprehensive genomic characterization of squamous cell lung cancers. Nature.

[9] Cancer Genome Atlas Research Network (2014). Comprehensive molecular profiling of lung adenocarcinoma. Nature.

[10] Labbé C, Cabanero M, Korpanty GJ (2017). Prognostic and predictive effects of TP53 co-mutation in patients with EGFR-mutated non-small cell lung cancer (NSCLC). Lung Cancer.

[11] Molina-Vila MA, Bertran-Alamillo J, Gascó A (2014). Nondisruptive p53 mutations are associated with shorter survival in patients with advanced non-small cell lung cancer. Clin Cancer Res.

[12] Zhao J, Han Y, Li J, Chai R, Bai C (2019). Prognostic value of KRAS/TP53/PIK3CA in non-small cell lung cancer. Oncol Lett.

[13] Gu J, Zhou Y, Huang L (2016). TP53 mutation is associated with a poor clinical outcome for non-small cell lung cancer: Evidence from a meta-analysis. Mol Clin Oncol.

[14] VanderLaan PA, Rangachari D, Mockus SM (2017). Mutations in TP53, PIK3CA, PTEN and other genes in EGFR mutated lung cancers: Correlation with clinical outcomes. Lung Cancer.

[15] Aggarwal C, Davis CW, Mick R, et al. Influence of TP53 Mutation on Survival in Patients With Advanced EGFRMutant Non-Small-Cell Lung Cancer. JCO Precis Oncol. 2018;2018:PO.18.00107. 10.1200/PO.18.00107. 10.1200/PO.18.00107 PMC6372114 30766968

[16] Canale M, Petracci E, Delmonte A (2017). Impact of TP53 mutations on outcome in EGFR-mutated patients treated with first-line tyrosine kinase inhibitors. Clin Cancer Res.

[17] Hou H, Qin K, Liang Y (2019). Concurrent TP53 mutations predict poor outcomes of EGFR-TKI treatments in Chinese patients with advanced NSCLC. Cancer Manag Res.

[18] Jin Y, Shi X, Zhao J (2018). Mechanisms of primary resistance to EGFR targeted therapy in advanced lung adenocarcinomas. Lung Cancer.

[19] Chen M, Xu Y, Zhao J (2019). Concurrent driver gene mutations as negative predictive factors in epidermal growth factor receptor-positive non-small cell lung cancer. EBioMedicine.

[20] Watanabe S, Yamaguchi OU, Masumoto AI (2018). Phase I study evaluating the combination of Afatinib with carboplatin and Pemetrexed after first-line EGFR-TKIs. Anticancer Res.

[21] Saito H, Fukuhara T, Furuya N (2019). Erlotinib plus bevacizumab versus erlotinib alone in patients with EGFR-positive advanced non-squamous non-small-cell lung cancer (NEJ026): interim analysis of an open-label, randomised, multicentre, phase 3 trial. Lancet Oncol.

[22] Hosomi Y, Morita S, Sugawara S (2020). Gefitinib alone versus Gefitinib plus chemotherapy for non-small-cell lung cancer with mutated epidermal growth factor receptor: NEJ009 study. J Clin Oncol.

[23] Wang Q, Gao W, Gao F, Jin S, Qu T, Lin F (2021). Efficacy and acquired resistance of EGFR-TKI combined with chemotherapy as first-line treatment for Chinese patients with advanced non-small cell lung cancer in a real-world setting. BMC Cancer.

[24] Yu PP, Vose JM, Hayes DF (2015). Genetic Cancer Susceptibility Testing: Increased Technology. Increased Complexity J Clin Oncol.

[25] Inamura K (2017). Lung Cancer: Understanding Its Molecular Pathology and the 2015 WHO Classification. Front Oncol.

[26] Detterbeck FC, Boffa DJ, Kim AW, Tanoue LT (2017). The eighth edition lung cancer stage classification. Chest.

[27] Therasse P, Arbuck SG, Eisenhauer EA, Wanders J, Kaplan RS, Rubinstein L (2000). New guidelines to evaluate the response to treatment in solid tumors. European Organization for Research and Treatment of Cancer, National Cancer Institute of the United States, National Cancer Institute of Canada. J Natl Cancer Inst.

[28] Li F, Du X, Zhang H (2017). Next-generation sequencing of Chinese stage IV lung cancer patients reveals an association between EGFR mutation status and survival outcome. Clin Genet.

[29] Jiao XD, Qin BD, You P, Cai J, Zang YS (2018). The prognostic value of TP53 and its correlation with EGFR mutation in advanced non-small cell lung cancer, an analysis based on cBioPortal data base. Lung Cancer.

[30] Poeta ML, Manola J, Goldwasser MA (2007). TP53 mutations and survival in squamous-cell carcinoma of the head and neck. N Engl J Med.

[31] Brosh R, Rotter V (2009). When mutants gain new powers: news from the mutant p53 field. Nat Rev Cancer.

[32] Muller PA, Vousden KH (2013). p53 mutations in cancer. Nat Cell Biol.

[33] Yang Z, Chen Y, Wang Y, Wang S, Hu M, Zhang B (2021). Efficacy of EGFR-TKI Plus chemotherapy or monotherapy as first-line treatment for advanced EGFR-Mutant Lung Adenocarcinoma Patients With Co-Mutations. Front Oncol.

[34] Li XM, Li WF, Lin JT, Yan HH, Tu HY, Chen HJ (2021). Predictive and prognostic potential of TP53 in patients with advanced non-small-cell lung cancer treated with EGFR-TKI: analysis of a Phase III randomized clinical trial (CTONG 0901). Clin Lung Cancer.

[35] Yoshimura N, Kudoh S, Mitsuoka S (2015). Phase II study of a combination regimen of gefitinib and pemetrexed as first-line treatment in patients with advanced non-small cell lung cancer harboring a sensitive EGFR mutation. Lung Cancer.

[36] Xu L, Qi Q, Zhang Y, Cui J, Liu R, Li Y (2019). Combination of icotinib and chemotherapy as first-line treatment for advanced lung adenocarcinoma in patients with sensitive EGFR mutations: a randomized controlled study. Lung Cancer.

[37] Han B, Jin B, Chu T (2017). Combination of chemotherapy and gefitinib as first-line treatment for patients with advanced lung adenocarcinoma and sensitive EGFR mutations: A randomized controlled trial. Int J Cancer.

[38] La Monica S, Madeddu D, Tiseo M (2016). Combination of Gefitinib and Pemetrexed prevents the acquisition of TKI Resistance in NSCLC Cell lines carrying EGFR-activating mutation. J Thorac Oncol.

[39] Takezawa K, Okamoto I, Tanizaki J (2010). Enhanced anticancer effect of the combination of BIBW2992 and thymidylate synthase-targeted agents in non-small cell lung cancer with the T790M mutation of epidermal growth factor receptor. Mol Cancer Ther.

[40] Cheng Y, Murakami H, Yang PC (2016). Randomized Phase II trial of Gefitinib with and without Pemetrexed as first-line therapy in patients with advanced nonsquamous non-small-cell lung cancer with activating epidermal growth factor receptor mutations. J Clin Oncol.

[41] Soria JC, Massard C, Le Chevalier T (2010). Should progression-free survival be the primary measure of efficacy for advanced NSCLC therapy. Ann Oncol.

[42] Hayashi H, Okamoto I, Morita S, Taguri M, Nakagawa K (2012). Postprogression survival for first-line chemotherapy of patients with advanced non-small-cell lung cancer. Ann Oncol.

[43] Goto K, Nishio M, Yamamoto N (2013). A prospective, phase II, open-label study (JO22903) of first-line erlotinib in Japanese patients with epidermal growth factor receptor (EGFR) mutation-positive advanced non-small-cell lung cancer (NSCLC). Lung Cancer.

[44] Fukuoka M, Wu YL, Thongprasert S (2011). Biomarker analyses and final overall survival results from a phase III, randomized, open-label, first-line study of gefitinib versus carboplatin/paclitaxel in clinically selected patients with advanced non-small-cell lung cancer in Asia (IPASS). J Clin Oncol.

[45] Inoue A, Kobayashi K, Maemondo M (2013). Updated overall survival results from a randomized phase III trial comparing gefitinib with carboplatin-paclitaxel for chemo-naïve non-small cell lung cancer with sensitive EGFR gene mutations (NEJ002). Ann Oncol.

[46] Shi YK, Wang L, Han BH (2017). First-line icotinib versus cisplatin/pemetrexed plus pemetrexed maintenance therapy for patients with advanced EGFR mutation-positive lung adenocarcinoma (CONVINCE): a phase 3, open-label, randomized study. Ann Oncol.

