# Supplementary material for: Efficacy and safety analyses of epidermal growth factor receptor tyrosine kinase inhibitors combined with chemotherapy in the treatment of advanced non–small-cell lung cancer with an EGFR/TP53 co-mutation
Source: BMC Cancer. 2022 Dec 12;22:1295. doi: 10.1186/s12885-022-10391-z (PMC9743525; doi:10.1186/s12885-022-10391-z)
Supplement: Supplementary file 1 — Additional file 1: Supplementary Table S1. Post-treatments after progression in T and TC group (n=95). Supplementary Table S2. TP53 mutations classified by missense or nonsense mutations; Supplementary Figure S1. TTP (A) and OS (B) curves of EGFR/TP53 co-mutation patients clarified by missense mutations or nonsense mutations. Supplementary Figure S2. TTP (A) and OS (B) curves of EGFR/TP53 co-mutation patients clarified by missense mutations or nonsense mutations in T group. TTP (C) and OS (D) curves of EGFR/TP53 co-mutation patients clarified by missense mutations or nonsense mutations in TC group. [file 12885_2022_10391_MOESM1_ESM.docx]

Supplementary Table S1. Post-treatments after progression in T and TC group (n=95)

|  | T group n (%) | TC group n (%) |
| --- | --- | --- |
| Second-line treatments |  |  |
| Osimertinib | 24(39.3) | 9(26.5) |
| Osimertinib + Chemotherapy | 3(4.9) | 2(5.9) |
| Chemotherapy | 8(13.1) | 3(8.8) |
| Comprehensive treatments including radiotherapy | 5(8.2) | 4(11.8) |
| Other Comprehensive treatments | 9(14.8) | 7(20.6) |
| unknow | 12(19.7) | 10(29.4) |

Comprehensive treatments: may be one of the following treatment options, EGFR-TKIs combined with chemotherapy or (and) antiangiogenic therapy, chemotherapy combined with immunotherapy or small molecule tyrosine kinase inhibitors combined with chemotherapy or immunotherapy.

Supplementary Table S2. TP53 mutations classified by missense or nonsense mutations

|  | T group n (%) | TC group n (%) |
| --- | --- | --- |
| Missense mutations | 43(70.5) | 26(76.5) |
| Nonsense mutations | 16(26.2) | 7(20.6) |
| NA | 2(3.3) | 1(2.9) |


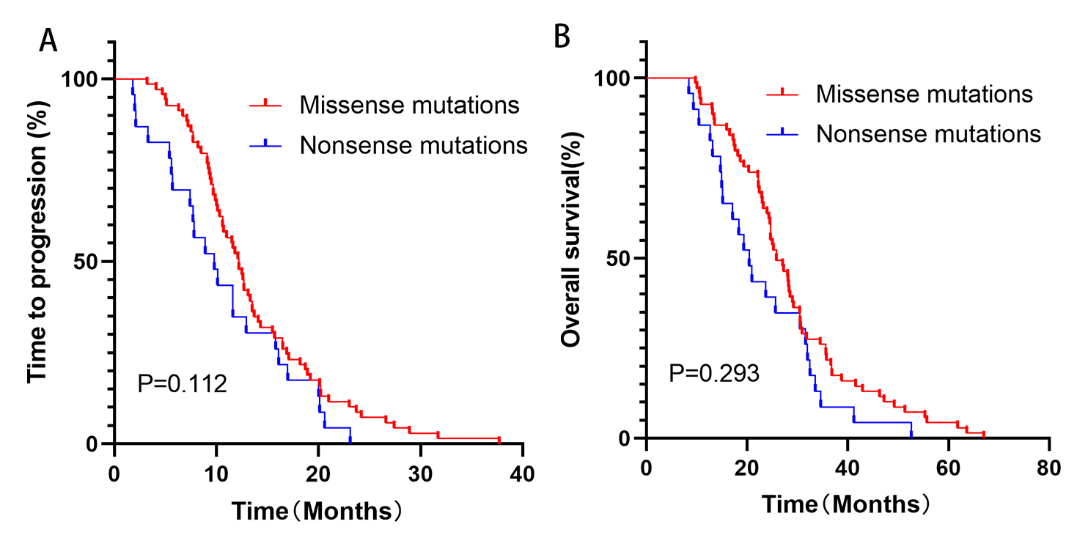


Supplementary Figure S1. TTP (A) and OS (B) curves of EGFR/TP53 co-mutation patients clarified by missense mutations or nonsense mutations.


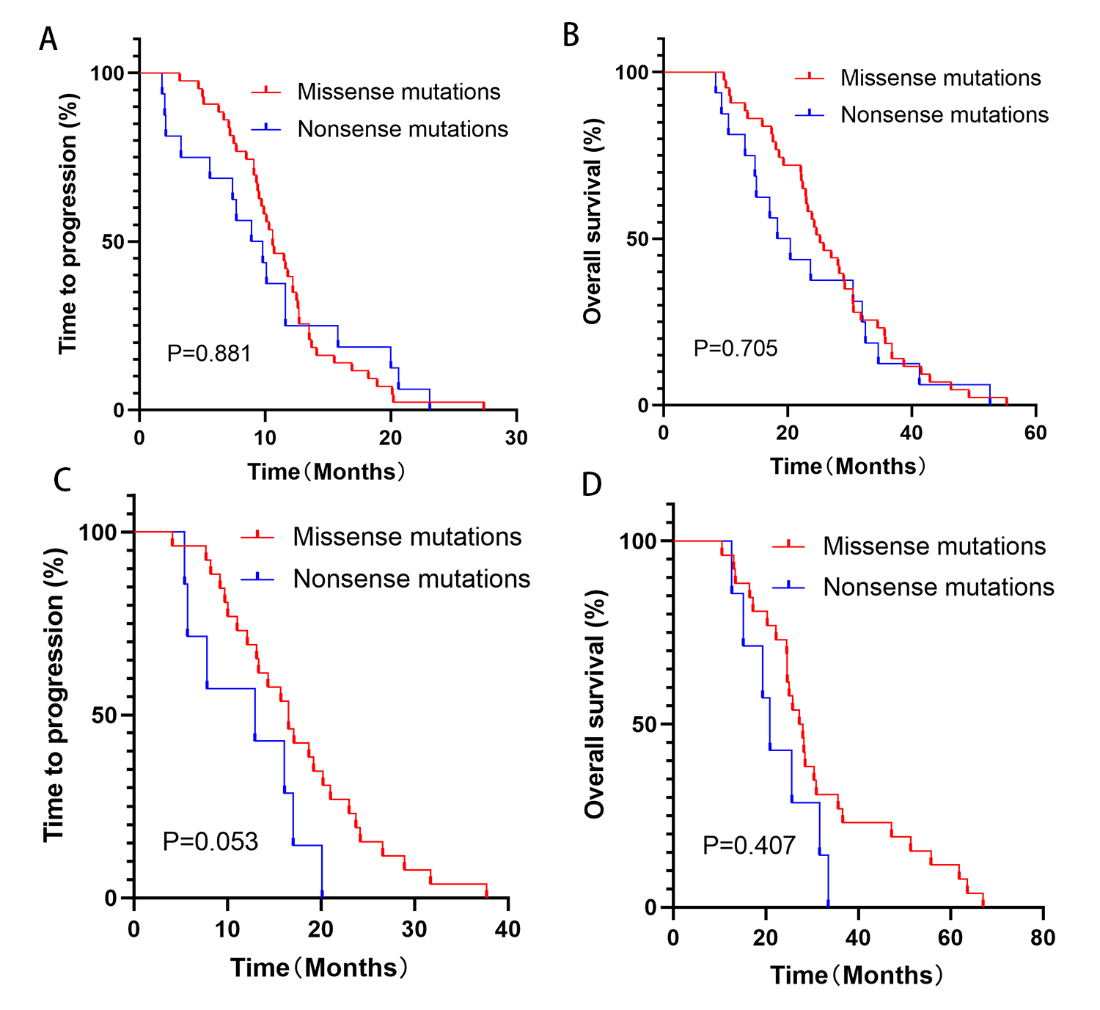


Supplementary Figure S2. TTP (A) and OS (B) curves of EGFR/TP53 co-mutation patients clarified by missense mutations or nonsense mutations in T group. TTP (C) and OS (D) curves of EGFR/TP53 co-mutation patients clarified by missense mutations or nonsense mutations in TC group.
